# Body Dissatisfaction, Eating Styles, Weight-Related Behaviors, and Health among Young Women in the United States

**DOI:** 10.3390/nu14183876

**Published:** 2022-09-19

**Authors:** Kaitlyn M. Eck, Virginia Quick, Carol Byrd-Bredbenner

**Affiliations:** 1 Department of Nutrition and Dietetics, Marywood University, Scranton, PA 18509, USA; 2 Department of Nutritional Sciences, School of Environmental & Biological Sciences, Rutgers, The State University of New Jersey, New Brunswick, NJ 08901, USA

**Keywords:** body dissatisfaction, maladaptive eating, adaptive eating, addictive eating, dichotomous thinking in eating, food neophobia, emotional eating, routine restraint, compensatory restraint, intuitive eating, mindful eating

## Abstract

Body dissatisfaction is a common condition that poses health behavior risks, such as the use of maladaptive eating styles instead of adaptive eating styles. Few studies have simultaneously examined both adaptive and maladaptive eating styles and their association with body dissatisfaction in a comprehensive manner. To address this gap, this study examined how body dissatisfaction is related to an array of adaptive and maladaptive eating styles, weight-related behaviors, and health status as well as the associations of health status, BMI, and weight-related behaviors with body dissatisfaction in 261 young adult women. Maladaptive eating styles, such as emotional eating, tended to rise in tandem with body dissatisfaction, differing significantly among body-dissatisfaction levels with medium to large effect sizes. For adaptive eating styles, as body dissatisfaction increased, compensatory restraint increased, intuitive eating declined, and mindful eating did not differ. Weight-related dietary, physical activity, and sleep behaviors did not differ by body dissatisfaction level. BMI increased and health status decreased as body dissatisfaction increased. Binary logistic regression revealed those who were body-dissatisfied had significantly lower health status, higher BMIs, and did not differ on weight-related behaviors. Study findings suggest strategies to improve health-promotion interventions aiming to increase body satisfaction.

## 1. Introduction

Body dissatisfaction—a negative self-assessment of one’s own physical appearance—is so commonly reported that some researchers have referred to it as a “normative” or “endemic” condition [1,2,3,4,5]. Evidence indicates that body dissatisfaction occurs across genders, across race/ethnic groups, and in all adult age groups. Women are more affected by body dissatisfaction than men, with prevalence rates ranging from 13 to 32% versus 9 to 28% for men depending on the assessment method used [1,6,7,8]. A recent study conducted in the U.S. reported little difference in body dissatisfaction prevalence by race/ethnicity [1], however a meta-analysis of some older research found African American adults were more satisfied with their bodies than white counterparts [9]. Young adulthood has been identified as a time when body dissatisfaction is particularly high [10,11,12] and tends to persist throughout adulthood [1].

Significant research suggests that body dissatisfaction is the result of social comparisons (i.e., comparing oneself to others to determine his or her status or rank on certain appearance dimensions) such as when comparing oneself to stylized, manipulated photographs of celebrities that cultivate and perpetuate culturally defined body shape ideals [13,14,15,16,17,18]. These observed societal “ideals” are then internalized; satisfaction or dissatisfaction with appearance is a function of the extent to which an individual perceives he or she matches those standards [19]. Idealized female body shapes tend to be slender and toned, whereas idealized male shapes tend to be muscular and toned. These shape ideals are highly dependent on body mass index (BMI), thus it is not surprising that body dissatisfaction tends to rise with BMI [1,8] or that body-shape dissatisfaction and body-weight dissatisfaction are highly correlated [20]. Indeed, perception of one’s body shape is a key determinant of body dissatisfaction [21,22,23,24].

Body dissatisfaction poses risks to physical and mental health. For instance, those with high body dissatisfaction are less likely to engage in health-protective behaviors, such as participating in cancer screening activities, meeting dietary recommendations, and engaging in exercise [25,26,27,28,29]. Those with greater body dissatisfaction also are more likely to endorse unhealthy behaviors and report that they have poor health status, smoke, sleep poorly, are interested in elective cosmetic surgery, and use unhealthy weight-control strategies (e.g., vomiting, laxatives, diet pills) [30,31,32,33,34,35]. Body dissatisfaction is associated with negative affect, including low self-esteem and greater anxiety and depression [7,8,36,37,38,39,40,41,42,43,44,45]. Depression is one of the strongest risk factors for maladaptive eating styles and can evolve into life-threatening eating disorders [46,47,48].

Maladaptive eating styles interfere with consuming a healthy diet and include emotional eating, restraint eating, food addiction, food neophobia, and unhealthy weight-management behaviors [49,50,51]. Emotional eating is provoked by feelings that cause individuals to use food as a strategy for coping [52]. Negative and uncomfortable emotions, such as stress, anxiety, or depression commonly trigger emotional eating [52], but in some cases, positive emotions such as confidence, happiness, and relaxation also can result in emotional eating [53]. Restraint eating is persistent, deliberate restriction of food intake and is often governed by self-imposed strict eating ”rules”, such as dichotomizing foods into “good” or “bad” groups then subsequently restricting intake to “good” foods [54,55]. When these “rules” are too inflexible or perturbed (e.g., succumbing to temptation to eat a “bad” food), individuals may lose control and engage in uncontrolled (binge) eating [54,55,56,57,58]. Restraint eaters tend to cycle through periods of control followed by periods of uncontrolled eating [55,59]. Food addiction is characterized as engaging in abnormal patterns of excessive food intake, experiencing withdrawal symptoms when the desired food is not eaten, and feeling regret over these behaviors [60,61]. Food neophobia is the reluctance to eat unfamiliar or new foods and is associated with reduced diet quality and eating disorders [62,63]. Unhealthy weight management behaviors are closely related to maladaptive eating because they are misperceived to be effective methods for controlling weight gain—often an undesired effect of maladaptive eating [56,64,65,66].

In contrast to maladaptive eating styles, adaptive eating styles support consumption of a healthy diet and are inversely related to body dissatisfaction [29,41,49,67]. Intuitive eating, mindful eating, and compensatory restraint eating are types of adaptive eating. Intuitive eating is regulating food intake by appropriately responding to physical cues of hunger and satiety [41,68]. Mindful eating includes being aware of the sensations of eating and paying attention to the act of eating while eating [69,70]. Compensatory restraint eating is consciously balancing intake at one meal in the day to compensate for higher-than-normal intake that occurred at an earlier meal in the day or will occur at a meal later in the day [71]. Adaptive eating styles are related to better weight management [67,72].

Despite widespread interest in body dissatisfaction, few studies have simultaneously examined both adaptive and maladaptive eating styles and their association with body dissatisfaction in a comprehensive manner, with most focusing on highly disordered eating behaviors, such as those associated with bulimia nervosa and anorexia nervosa, or examining just one or two eating styles. Thus, the first aim of this study was to examine how body dissatisfaction is related to an array of adaptive and maladaptive eating styles, weight-related behaviors, and health status. The second aim of this study was to examine associations of health status, BMI, and weight-related behaviors (i.e., diet, physical activity, sleep) with body dissatisfaction. The target audience was young adult women given that during this life stage, women’s eating styles associated with body dissatisfaction have been documented to increase and the health behaviors established during this time period tend to persist into adulthood and predict long-term health status [37,73,74,75,76]. It was hypothesized that greater body dissatisfaction would be associated with less use of adaptive and more use of maladaptive eating styles, as well as poorer health status and unhealthier BMIs and weight-related behaviors. A greater understanding of these interrelationships could highlight important ways to improve health promotion interventions.

## 2. Materials and Methods

This cross-sectional study was approved by the Institutional Review Board (IRB) at the authors’ university. Participants gave informed consent prior to participation in the online survey by clicking the “agree to participate” button.

### 2.1. Sample

The sample was recruited to complete an online survey about student health via an email sent to official university listservs. The recruitment notices indicated the survey would take about 30 minutes to complete and participants would have the chance to win a drawing of 1 of 5 $25 gift cards. Eligibility criteria set for this secondary analysis were being female, between 18 and 26 years of age, having completed high school in the United States to control for sociocultural differences, and being enrolled as a full-time, undergraduate student at the large, public university in the northeastern United States where the study was conducted. 

### 2.2. Instrument

Data were gathered using Qualtrics^®^ Survey Software. The survey collected demographic characteristics (i.e., age, race/ethnicity) and assessed several eating styles, weight-related behaviors, and health status. A panel of 6 experts in nutrition and eating behaviors identified the maladaptive and adaptive eating styles to be studied and the valid, reliable questionnaires for assessing eating styles and body satisfaction used in this study. Maladaptive measures included addictive eating, dichotomous thinking in eating, food neophobia, emotional eating, routine restraint, and unhealthy eating behaviors [51,70,77,78,79]. Adaptive eating styles assessed included compensatory restraint, intuitive eating, and two components of mindful eating: awareness and attention [70,77,78].

The Eating Disorder Examination Questionnaire (EDE-Q) Body Shape Concerns item (i.e., “During the past 28 days, how dissatisfied have you been with your body shape?”) assessed body dissatisfaction [80,81]. Response choices were not at all, slightly, somewhat, moderately, and a lot, scored 1 to 5, respectively. Higher scores indicate greater dissatisfaction with one’s body shape.

#### 2.2.1. Maladaptive Eating Instruments

The brief Yale Food Addiction Scale measured addictive-like eating behaviors using 4 items (e.g., “I have had withdrawal symptoms such as agitation, anxiety, or other physical symptoms when I cut down or stopped eating certain foods”). Answer choices were on a 5-point frequency scale (i.e., never, once a month, 2 to 4 times a month, 2 to 3 times a week, 4 or more times a week) [82,83,84].

The Dichotomous Thinking in Eating scale evaluated the extent to which individuals applied rigid, “all or nothing” or “black and white” rules as it relates to food choices (e.g., “I think of food as good or bad”). This 2-item scale had 5-point agreement answers ranging from strongly disagree to strongly agree [85]. 

Food neophobia is reluctance to eat unfamiliar foods. This eating style was assessed using the 2-item Food Neophobia scale (e.g., “I am afraid to eat things I have never eaten before”) [86,87]. Items were answered using 5-point agreement answers ranging from strongly disagree to strongly agree.

Emotional eating, routine restraint, and compensatory restraint were assessed using the Weight-Related Eating Questionnaire (WREQ) [71]. The Emotional Eating scale uses 5 items to assess the tendency to respond to negative emotions by eating (e.g., “I tend to eat when I am disappointed or feel let down”) [88]. The Emotional Eating scale contains 3 subscales measuring reasons for engaging in emotional eating: stress-induced, depression-induced, and relationship-discord-induced. Routine restraint is constantly restricting dietary intake to control weight (e.g., “I purposely hold back at meals in order not to gain weight”). The Routine Restraint scale contained 3 items. Compensatory restraint is controlling dietary intake at one meal to balance overall intake to match needs; the focus is on regulating intake rather than persistently restricting intake [67]. This may mean eating a lighter dinner when a larger-than-usual meal was eaten earlier in the day, or eating somewhat less at lunch when planning to eat a larger dinner meal, such as at a party or restaurant. An example item from this 3-item scale is “If I eat more than usual during a meal, I try to make up for it by eating less at another meal”. All WREQ items asked participants to indicate how much each item described them using 5-point frequency answer choices ranging from “not at all” to “completely”.

Use of unhealthy weight-control methods was assessed by 9 yes/no items identified from the literature, expert review, and adapted from many sources (such as Fichter et al. [89]) to increase the comprehensiveness of methods used. Participants indicated whether they used these methods to lose weight or prevent weight gain in the past year: fasting, food restriction/dieting, diet pills, vomiting, laxatives, diuretics, food substitutes (powders, drinks), skipped meals, and smoking. All yes answers were awarded 1 point and no answers 0 points. The scale score was a sum of response scores.

All other maladaptive-eating-styles scales had answer choices that were on 5-point scales, which were scored 1 to 5. Responses to each item on a scale were averaged to create a scale score. Thus, scores for maladaptive-eating-styles scales could range from 1 to 5, with higher scores indicating greater expression of the eating style. 

#### 2.2.2. Adaptive Eating Instruments

Intuitive eating is eating in response to physiological signals [78]. That is, eating when hungry and stopping when satiated. The Hunger and Satiety subscale from the Intuitive Eating scale assessed intuitive eating with 3 items (e.g., “I trust my body to tell me when to stop eating”) that were answered using 5-point agreement answer choices ranging from strongly disagree to strongly agree [68].

Mindful eating is drawn from the concept of “mindfulness” which is commonly used to describe the mental processes of conscious awareness and consistent attention to a current situation [90,91,92,93]. The Awareness and Attention (sometimes stated in the converse, Distraction) subscales of the Mindful Eating Questionnaire measured two domains of mindful eating [69]. The Mindful Eating: Awareness scale consisted of 3 items evaluating the frequency with which individuals were cognizant of the physical characteristics (e.g., color, scent, flavor) of foods while eating (e.g., “I notice when there are subtle flavors in the foods I eat”) [69]. The 2-item Mindful Eating: Attention scale evaluated how frequently individuals consciously stayed focused during the act of eating (e.g., “I think about other things I need to do while I am eating”) [69]. Items on both mindful eating scales were answered using 5-point frequency choices ranging from almost never to almost always. 

All adaptive-eating-styles scales were answered in the same manner as the maladaptive-eating-style scales. That is, all had 5-point answer choices that were scored from 1 to 5 (reversed in the case of the inversely stated Mindful Eating: Attention scale items). All responses to items on a scale were averaged to generate mean scores. Accordingly, scores for adaptive-eating-styles scales could range from 1 to 5; higher scores indicate greater expression of the eating style. 

#### 2.2.3. Weight-Related Behavior Assessments

Five weight-related behaviors were assessed: fruit/vegetable intake, fat intake, sugar-sweetened beverage intake, physical activity, and sleep duration. The 7-item Block Fruit/Vegetable Screener and 17-item Block Fat Screener were used to determine daily servings of fruits and vegetables and percent total calories from fat, respectively [94,95,96,97]. Daily serving amount of sugar-sweetened drinks was determined using the HOMES Sugar-Sweetened Beverage questionnaire [98]. The HOMES Physical Activity questionnaire, which uses days/week of engaging in walking, moderate physical activity, and healthy physical activity for at least 10 min at a time, was used to estimate physical activity level, with possible scores ranging from 0 to 42 [98,99,100]. The Pittsburgh Sleep Quality Index (PSQI) 1-item sleep duration component measured total hours of sleep nightly [101,102]. 

#### 2.2.4. Health Status Assessments

Health status was assessed using the Centers for Disease Control and Prevention Health Quality of Life questionnaire (i.e., general health, days of “not good” physical and mental health in the past month) [103,104]. BMI was calculated from self-reported height and weight using the standard formula [105].

### 2.3. Data Analysis

To examine how body dissatisfaction is related to eating styles, weight-related behaviors, and health status, participants were stratified by their responses to the EDE-Q Body Shape Concerns item into 5 comparison groups (i.e., those who were not at all, slightly, somewhat, moderately, and a lot dissatisfied). Descriptive statistics (i.e., means, standard deviations, 95% confidence intervals) were computed for each body-dissatisfaction group for demographic characteristics and maladaptive and adaptive eating styles. Analysis of variance (ANOVA) and Tukey post-hoc procedures were performed to determine whether any assessment differed by body dissatisfaction group. Significance was set at *p* ≤ 0.05. Partial eta-squared values were determined to express effect size of significant differences; standard thresholds for small, medium, and large effect sizes (i.e., 0.01, 0.06, and 0.14, respectively) were applied [106]. 

Subsequent analyses examined associations of health status, BMI, and weight-related behaviors of participants who were and were not satisfied with their body shape using binary logistic regression. Participants were dichotomized into two groups using responses to the EDE-Q Body Shape Concerns (i.e., those who were not at all or slightly dissatisfied were classified as body satisfied, whereas those who were moderately or a lot dissatisfied were categorized as body dissatisfied). Binary logistic regression data were expressed as odds ratios (OR) and 95% confidence intervals. Analyses were conducted using the Statistical Package for Social Sciences (SPSS) version 28 (IBM, Chicago, IL, USA). 

## 3. Results

### 3.1. Participant Characteristics

The young adult women college students (N = 261) who participated in this study averaged about 20 years of age and 50% were white (Table 1). When categorized by body dissatisfaction level, 9%, 31%, 20%, 19%, and 21% had no, low, low–moderate, moderate, and high body dissatisfaction, respectively. Analysis of variance revealed no significant differences in age or race/ethnicity (white vs. non-white) by body dissatisfaction level.

### 3.2. Maladaptive Eating Styles

As shown in Table 2, mean scores on the maladaptive-eating-styles scales tended to rise in tandem with increasing body dissatisfaction level. Except for the Food Neophobia scale, maladaptive eating styles differed significantly among the body-dissatisfaction levels with large effect sizes for all scales except Dichotomous Eating which had a medium effect size. The no, low, and low–moderate groups tended to have significantly lower mean scores on the Food Addiction scale than the moderate- and high body dissatisfaction groups. All body dissatisfaction groups scored significantly lower on the Dichotomous Eating scale than the high body dissatisfaction group. Nearly all pairwise comparisons differed significantly on the Emotional Eating scale, with stress, depression, and relationship discord all inducing emotional eating similarly. Routine Restraint mean scores increased with body dissatisfaction level, with the moderate and high body dissatisfaction groups scoring significantly higher than those with no or low body dissatisfaction. Those with moderate and high body dissatisfaction levels tended to use significantly more unhealthy weight-control methods than those with less body dissatisfaction.

### 3.3. Adaptive Eating Styles

Adaptive eating style scale results indicate that Compensatory Restraint mean scores rose with body dissatisfaction whereas Intuitive Eating mean scores were inversely related to body dissatisfaction level. The moderate- and high-body-dissatisfaction groups tended to differ significantly from the no and low body dissatisfaction groups on the Compensatory Restraint and Intuitive Eating scales. No significant differences by body dissatisfaction level were noted for either the Mindful Eating: Awareness or Mindful Eating: Attention scales.

### 3.4. Weight-Related Behaviors

Body-dissatisfaction groups did not differ significantly in their intake of fruits and vegetables, however those with no or low dissatisfaction consumed more daily servings than comparison groups. Percent of total calories from fat varied little across body dissatisfaction levels and did not differ significantly. Sugar-sweetened beverage intake also did not differ significantly among body dissatisfaction groups, however those in the high body dissatisfaction group consumed the most servings daily. Physical activity level was highest in the no dissatisfaction group and lowest in the high dissatisfaction group, with these two groups differing significantly. 

### 3.5. Health Status

Overall health status was inversely related to body dissatisfaction, with those in the no and low groups having significantly better health than those with more dissatisfaction. Total days of “not good” physical health and mental health tended to rise with increasing body dissatisfaction. The no and low groups tended to have significantly fewer days of “not good” health than those with more body dissatisfaction.

### 3.6. Binary Logistic Regression Findings

The no and low body dissatisfaction groups did not differ significantly on any of the eating styles scales. The moderate and high body dissatisfaction groups differed only on the Dichotomous Eating scale. Given their similarities in the use of maladaptive and adaptive eating styles, the no and low body dissatisfaction groups were combined to form the body satisfied group (*n* = 106) and the moderate and high groups were combined to form the body dissatisfied group (*n* = 104) in order to compare how these two groups differed with regard to health status, BMI, and weight-related behaviors (Table 3). Binary logistic regression analyses revealed the body dissatisfied group had significantly (*p* < 0.05) lower general health status (OR = 0.48 [95% CI 0.35–0.65]), more days of “not good” physical (OR = 1.09 [95% CI 1.03–1.15]) and mental (OR = 1.06 [95% CI 1.02–1.10]) health in the past month, and higher BMIs (OR = 1.36 [95% CI 1.22–1.51]) than the body satisfied group (Table 3). There were no significant body dissatisfaction and satisfaction group differences for fruit and vegetable intake, sugar-sweetened beverage intake, physical activity level, and sleep duration.

## 4. Discussion

The findings of this study indicate that body dissatisfaction is related to greater use of all types of maladaptive eating styles studied, except food neophobia. Among the adaptive eating styles investigated, mindful eating was not related to body dissatisfaction. However, intuitive eating was used less as body dissatisfaction increased whereas the opposite was true for compensatory restraint. Dietary intake and sleep duration behaviors were unrelated to body dissatisfaction, whereas physical activity declined with increasing dissatisfaction. Although measures of health status tended to decline as body dissatisfaction increased, BMI was positively correlated with body dissatisfaction. In the subset of participants dichotomized into body satisfied and body dissatisfied categories, binary logistic regression revealed the body dissatisfied group had significantly poorer health status, more physically and mentally unhealthy days in the past month, and higher BMIs than the body satisfied group. 

As hypothesized, body dissatisfaction was significantly associated with all maladaptive eating styles, except food neophobia. These associations likely relate to sociocultural pressures to be thin promoted by a variety of sources in Western culture, such as mass media, parents, and friends [107,108]. Sociocultural pressures and persistent messages to be thin may, over time, lead to a heightened level of internalization of the thin “ideal” and, thus, foment and perpetuate body dissatisfaction [109]. In turn, body dissatisfaction may increase the risk for maladaptive eating behaviors as a perceived mechanism to control one’s body weight and shape [88,110].

Restraint eating is generally defined in a unidimensional manner as being persistent or routine calorie restriction, and rarely considers that restraint eating also can be expressed in a more positive way to balance overall calorie intake on an “as needed” basis to compensate for infrequent occasions of higher than usual food consumption. To consider both dimensions of restraint eating, the current study used the WREQ to distinguish between those who have a maladaptive restraint eating pattern (routine restraint) and those that have a more flexible, adaptive approach to weight control characterized by episodic intentional caloric restriction to offset overconsumption at one meal (compensatory restraint) [71]. Interesting, both compensatory restraint eating and routine restraint were positively associated with body dissatisfaction. It is not clear why compensatory restraint was higher in those with more body dissatisfaction than in their more satisfied counterparts. Body dissatisfaction may be so pervasive in the Western society that it has become normative to be restrictive in one’s food intake both routinely and in compensation for episodically larger meals. Future research should investigate this finding further. Additionally, cognitive testing of the WREQ with those of varying levels of body dissatisfaction could provide insights into how body dissatisfaction level may affect the interpretation of the questionnaire items and may highlight needed adjustments to increase the clarity of the items.

Adoption of maladaptive eating behaviors and associated negative emotions like depression and anxiety can severely impact physical and psychosocial health [111,112]. Thus, it is logical that emotional eating, regardless of whether it was induced by stress, depression, or relationship discord, was significantly associated with body dissatisfaction. Prior work has found that emotional dysregulation (i.e., difficulty in receiving, processing, and displaying emotions and lack of adaptive coping with stress) mediates the relationship between emotional eating and internalization of weight bias [113]. However, there is some evidence that higher levels of mindfulness are associated with greater awareness of eating patterns and a lower level of stress and incidence of emotional eating [114]. 

Although mindful eating may be effective in the treatment of body dissatisfaction, unhealthy eating patterns, and emotional dysregulation [115,116,117], the current study’s findings suggest that the attention and awareness components of mindful eating are not associated with body dissatisfaction. One cross-sectional study reported that the relationship between body dissatisfaction and mindful eating was stronger in overweight and obese subjects than normal weight participants [118]. Most participants in this current study were of normal body weight, which may at least partially explain the non-significant associations found between mindful eating and body dissatisfaction. 

On the other hand, as hypothesized, intuitive eating was significantly associated with body dissatisfaction. That is, those with lower body dissatisfaction endorsed more intuitive eating, an adaptive eating style, compared to those with high body dissatisfaction. Others also have reported that intuitive eating is associated with lower levels of body dissatisfaction along with less disordered eating and psychological distress [119,120,121,122]. 

Future work is warranted in further exploring the use of mindful eating and intuitive eating approaches for the prevention and treatment of negative body image.

The hypothesis that body dissatisfaction would be significantly associated with higher BMIs and poorer health status was supported in the binary logistic regression analyses. Findings align with the literature documenting that greater BMI and body weight are commonly associated with body dissatisfaction [23,123]. The current study also lends support to findings reported by Durkin and Paxton demonstrating that body dissatisfaction predicted more days of “not good” health among college students in the United States [124]. Additionally, findings are congruent with a population-based study of adults that reported positive body image was a predictor of health quality of life [125].

The hypothesis that body dissatisfaction would be significantly associated with lower fruit/vegetable intake, higher percent of total calories from fat, higher daily sugar-sweetened beverage intake, lower physical activity level, and shorter sleep duration was not supported in the binary logistic regression analyses. There are few other studies that have investigated body dissatisfaction and weight-related behaviors, and their results are mixed. In a cross-sectional study of Polish adolescents, those who were body dissatisfied met dietary recommendations less often than their satisfied counterparts and were less likely to meet vegetable intake recommendations; however, both groups had similar intakes of fruit, whole grain, sweet beverages, and fast food [26]. A study of adults living in the United States also found no difference in dietary quality (based on intake of fruit, vegetables, whole grains, and calcium) among those with positive vs. negative body image [126]. Results of research involving university students in India revealed no links between physical activity and body dissatisfaction [23]. In contrast, body dissatisfaction predicted decreased physical activity among college students in the United States [124]. No studies of sleep duration and body dissatisfaction could be located, however de Sousa Matias et al. reported that body dissatisfaction was associated with impaired sleep quality among a population-based sample of Brazilian adolescents [30]. Given that poor-quality sleep is known to shorten sleep duration [127], it is likely these youth also had shorter sleep, but whether it would have differed significantly, as was not the case in the current study, remains unknown. 

The variation in results among studies may be due to the nature of how body dissatisfaction was measured [128]. For instance, body dissatisfaction measures used in research include those assessing preoccupation with physical appearance, importance of and time devoted to physical appearance, drive for muscularity, discrepancy between actual and perceived body shape, as well as degree of dissatisfaction as was used in this study [128]. 

To our knowledge, this is the first study to comprehensively examine how body dissatisfaction is related to both adaptive and maladaptive eating styles as well as weight-related behaviors and health status. Additionally, it is the first to examine two dimensions of restraint eating vis à vis body dissatisfaction and to investigate distinct emotions that may induce emotional eating. Study strengths include the use of valid, reliable instruments for assessing all study variables. Given the sensitive nature of some of the survey items, such as addictive eating behaviors, the online administration of the survey is a strength in that this mode of data collection affords greater privacy and increases the likelihood of unbiased, socially desirable responses [129,130]. Height and weight were self-reported and may be subject to over- or under-reporting, however research findings suggest strong concordance of self-reported vs. objectively measured height and weight in young adult college students [131]. Like all secondary data analyses, this study is limited to the existing data. Men were not included in this study and are an important target for future research due to the growing reports of increasing body dissatisfaction in this audience [1,6]. The cross-sectional nature of this research limits the ability to draw cause-and-effect conclusions; future research should consider the temporal associations among body dissatisfaction and eating styles, health status, and behaviors.

## 5. Conclusions

The results of this study suggest that future interventions aiming to promote body satisfaction and appreciation should address the array of maladaptive eating styles those who are dissatisfied may adopt, offering instruction on how to alter patterns to use adaptive eating styles instead. For instance, participant use of mindful eating did not differ, yet studies indicate this adaptive eating style helps individuals to manage intake and become more aware of physiological signals of hunger and satiety, thereby enabling intuitive eating and moderating maladaptive eating behaviors [132,133,134,135,136,137].

The use of compensatory restraint by those with higher levels of body dissatisfaction should be encouraged. This can help individuals with weight dissatisfaction use this method of calorie control instead of routine restraint, which can result in rebound eating [54,55,56,57,58]. Application of study findings to health-promotion programs indicates an opportunity to promote adaptive eating styles as a substitute for maladaptive eating styles and as strategies for controlling weight and undergirding body satisfaction. The findings of this study make a significant contribution to the literature and provide insights into intervention content and strategies. The prevalence of body dissatisfaction, and its potentially devasting consequences, warrants the development of public health interventions that promote body satisfaction and appreciation.

## Figures and Tables

**Table 1:** Associations of Demographic Characteristics by Body Shape Dissatisfaction Level Among Young Adult Women College Students (N = 261).

Body Shape Dissatisfaction Level							
	None (*n* = 24)	Low (*n* = 82)	Low–Moderate (*n* = 51)	Moderate (*n* = 49)	High (*n* = 55)		
Characteristic	Mean ± SD (95% CI *) or N (%)	Mean ± SD (95% CI *) or N (%)	Mean ± SD (95% CI *) or N (%)	Mean ± SD (95% CI *) or N (%)	Mean ± SD (95% CI *) or N (%)	*F*	ANOVA † *p*
**Age (years)**	20.05 ± 1.77 (19.58–20.53)	20.01 ± 1.75 (19.71–20.30)	20.13 ± 1.75 (19.73–20.54)	19.88 ± 1.46 (19.51–20.24)	20.05 ± 2.10 (19.53–20.56)	0.193	0.942
**Race/Ethnicity ^1^**	0.53 ± 0.50 (0.39–0.66)	0.46 ± 0.50 (0.38–0.54)	0.56 ± 0.50 (0.45–0.67)	0.40 ± 0.49 (0.28–0.52)	0.61 ± 0.49 (0.49–0.73)	1.932	0.104
White	11 (46%)	41 (50%)	22 (43%)	33 (67%)	23 (42%)		
Asian (e.g., Japanese, Chinese, Korean)	3 (13%)	14 (17%)	7 (14%)	5 (10%)	11 (20%)		
Asian Indian	4 (17%)	5 (6%)	6 (12%)	3 (6%)	6 (11%)		
Black	1 (4%)	6 (7%)	6 (12%)	3 (6%)	4 (7%)		
Latino	2 (8%)	10 (12%)	6 (12%)	2 (4%)	9 (16%)		
Other (Mixed race, Pacific Islander)	3 (13%)	6 (7%)	4 (8%)	3 (6%)	2 (4%)		

* CI = Confidence Interval. † Analysis of Variance (ANOVA) for continuous variables (df = 4256). ^1^ Dichotomous Scoring of Race/Ethnicity groups into White = 0 and Non-white = 1.

**Table 2:** Associations of Eating of Styles, Weight-Related Behaviors, and Health Status with Body Dissatisfaction Level Among Young Adult Women College Students (N = 261).

Body Shape Dissatisfaction Level #								
	None (*n* = 24)	Low (*n* = 82)	Low– Moderate (*n* = 51)	Moderate (*n* = 49)	High (*n* = 55)	*F* † (*p*-Value)	Between- Group Differences ‡	Partial Eta- Squared
Characteristic	Mean ± SD (95% CI *)	Mean ± SD (95% CI *)	Mean ± SD (95% CI *)	Mean ± SD (95% CI *)	Mean ± SD (95% CI *)			
**Maladaptive Eating**								
Food Addiction ^1^	1.46 ± 0.73 (1.27–1.66)	1.44 ± 0.57 (1.34–1.53)	1.71 ± 0.74 (1.54–1.88)	1.99 ± 0.81 (1.79–2.19)	2.22 ± 0.95 (1.98–2.45)	16.288 (<0.0001)	C,D,F,G,I	0.189
Dichotomous Eating ^2^	3.31 ± 0.93 (3.06–3.56)	3.19 ± 0.81 (3.05–3.33)	3.31 ± 1.03 (3.08–3.55)	3.41 ± 0.79 (3.21–3.60)	3.89 ± 0.91 (3.66–4.11)	7.173 (<0.001)	D,G,I,J	0.086
Food Neophobia ^3^	2.15 ± 1.06 (1.87–2.44)	2.09 ± 0.98 (1.93–2.26)	2.28 ± 1.10 (2.03–2.53)	2.05 ± 1.00 (1.80–2.29)	2.20 ± 1.07 (1.94–2.47)	0.613 (0.653)	---	--
Emotional Eating ^4^	1.68 ± 0.80 (1.47–1.90)	1.94 ± 0.98 (1.77–2.10)	2.25 ± 1.06 (2.00–2.49)	2.83 ± 1.18 (2.54–3.12)	3.15 ± 1.36 (2.82–3.49)	22.621 (<0.0001)	B,C,D,F,G,H,I	0.249
Stress-Induced	1.96 ± 1.06 (1.68–2.25)	2.24 ± 1.15 (2.05–2.43)	2.71 ± 1.36 (2.40–3.03)	3.16 ± 1.31 (2.84–3.49)	3.45 ± 1.49 (3.09–3.82)	17.027 (<0.0001)	B,C,D,F,G,I	0.197
Depression-Induced	1.50 ± 0.75 (1.29–1.70)	1.74 ± 0.98 (1.57–1.90)	1.93 ± 0.99 (1.71–2.16)	2.61 ± 1.20 (2.31–2.91)	2.95 ± 1.42 (2.60–3.30)	22.563 (<0.0001)	C,D,F,G,H,I	0.248
Relationship-Discord-Induced	1.29 ± 0.15 (1.58–0.00)	1.50 ± 0.09 (1.68–0.00)	1.65 ± 0.12 (1.90–0.00)	2.29 ± 0.13 (2.56–0.00)	2.45 ± 0.13 (2.72–0.00)	15.560 <0.0001)	C,D,F,G,H,I	0.182
Routine Restraint ^5^	1.48 ± 0.85 (1.25–1.71)	1.71 ± 0.89 (1.56–1.86)	2.03 ± 0.91 (1.82–2.23)	2.18 ± 0.93 (1.95–2.41)	2.58 ± 1.18 (2.29–2.87)	14.068 (<0.0001)	B,C,D,F,G,I	0.167
Unhealthy Weight Control Methods ^6^	0.64 ± 1.44 (0.25–1.03)	0.74 ± 1.19 (0.54–0.94)	0.97 ± 1.22 (0.69–1.25)	1.58 ± 1.63 (1.18–1.99)	2.79 ± 2.06 (2.28–3.29)	25.806 (<0.0001)	C,D,F,G,I	0.275
**Adaptive Eating**								
Compensatory Restraint ^7^	1.77 ± 0.90 (1.53–2.01)	2.24 ± 1.19 (2.04–2.43)	2.52 ± 1.11 (2.26–2.77)	2.98 ± 1.12 (2.71–3.26)	3.02 ± 1.27 (2.71–3.33)	13.843 (<0.001)	B,C,D,F,G	0.164
Intuitive Eating ^8^	3.82 ± 0.86 (3.59–4.06)	3.76 ± 0.72 (3.64–3.89)	3.56 ± 0.77 (3.38–3.74)	3.19 ± 0.80 (3.00–3.39)	2.61 ± 0.99 (2.37–2.86)	27.710 (<0.001)	C,D,F,G,I	0.290
Mindfulness: Awareness ^9^	3.19 ± 0.88 (2.95–3.43)	3.25 ± 0.87 (3.11–3.40)	3.05 ± 0.87 (2.85–3.25)	3.43 ± 0.80 (3.23–3.63)	3.22 ± 1.06 (2.96–3.48)	1.638 (0.164)	--	--
Mindfulness: Attention ^10^	2.76 ± 0.89 (2.52–3.00)	2.59 ± 0.92 (2.44–2.74)	2.76 ± 0.93 (2.55–2.97)	2.52 ± 0.84 (2.31–2.72)	2.58 ± 1.19 (2.28–2.87)	0.961 (0.429)		--
**Weight-Related** **Behaviors**								
Fruit/Vegetable Intake, servings/day	4.32 ± 2.25 (3.71–4.93)	4.07 ± 1.99 (3.74–4.41)	3.58 ± 2.02 (3.12–4.05)	3.73 ± 2.05 (3.22–4.24)	3.99 ± 2.35 (3.42–4.57)	1.289 (0.274)	--	--
% Total Calories from Fat	34.31 ± 7.04 (32.41–36.22)	33.68 ± 6.37 (32.61–34.74)	31.99 ± 4.47 (30.96–33.02)	33.38 ± 5.18 (32.10–34.67)	34.70 ± 6.24 (33.17–36.24)	2.160 (0.073)	--	--
Sugar-Sweetened Beverages, servings/day	0.63 ± 0.72 (0.43–0.82)	0.59 ± 0.61 (0.48–0.69)	0.56 ± 0.60 (0.42–0.70)	0.55 ± 0.59 (0.40–0.69)	0.73 ± 0.86 (0.52–0.94)	0.822 (0.512)	--	--
Physical Activity Level ^11^	25.29 ± 14.89 (21.27–29.32)	20.19 ± 11.47 (18.27–22.12)	18.52 ± 11.61 (15.85–21.19)	19.51 ± 11.73 (16.60–22.42)	15.58 ± 10.25 (13.06–18.10)	5.284 (<0.001)	B,D	0.062
Sleep Duration, hours/night	6.49 ± 1.33 (6.13–6.85)	6.94 ± 1.38 (6.71–7.17)	6.48 ± 1.32 (6.18–6.78)	6.82 ± 1.75 (6.38–7.25)	6.39 ± 1.74 (5.97–6.82)	2.378 (0.051)	--	--
**Health Status**								
Overall Health Status	3.76 ± 1.07 (3.47–4.05)	3.69 ± 0.82 (3.55–3.83)	3.24 ± 0.97 (3.02–3.46)	3.02 ± 0.94 (2.78–3.25)	2.82 ± 1.02 (2.57–3.07)	14.760 (<0.0001)	B,C,D,E,F,G	0.188
Physical Health (days/month “not good” health)	3.02 ± 5.26 (1.60–4.44)	3.04 ± 3.91 (2.38–3.69)	4.83 ± 5.60 (3.54–6.11)	5.86 ± 6.65 (4.21–7.51)	6.03 ± 7.79 (4.12–7.94)	5.360 (<0.001)	D,F,G	0.063
Mental Health (days/month “not good” health)	6.42 ± 7.93 (4.27–8.56)	5.78 ± 6.27 (4.73–6.84)	7.37 ± 7.05 (5.75–9.00)	8.86 ± 7.88 (6.91–10.81)	12.02 ± 9.22 (9.75–14.28)	8.643 (<0.0001)	D,F,G,I	0.105
Body Mass Index	22.78 ± 2.83 (22.02–23.54)	22.14 ± 2.85 (21.66–22.62)	23.57 ± 4.33 (22.57–24.57)	24.64 ± 3.81 (23.70–25.59)	24.99 ± 5.15 (23.72–26.25)	8.981 (<0.0001)	D,F,G,I	0.109

* CI = Confidence Interval. # EDE-Q Body Shape Concerns question (i.e., “During the past 28 days, how dissatisfied have you been with your body shape?”) with higher mean scores indicating greater body dissatisfaction. † Analysis of Variance (ANOVA) with df = 256. ‡ Capital letters indicate significant (*p* < 0.05) Tukey post-hoc tests between group differences: A = None vs. Low; B = None vs. Low–Moderate; C = None vs. Moderate; D = None vs. High; E = Low vs. Low–Moderate; F = Low vs. Moderate; G = Low vs. High; H = Low–Moderate vs. Moderate; I = Low–Moderate vs. High; J = Moderate vs. High. ^1^ Yale Food Addiction Scale; Cronbach alpha = 0.74; 4 items; Answer choices: 5-point frequency scale (i.e., never, once a month, 2 to 4 times a month, 2 to 3 times a week, 4 or more times a week) [82,83,84]. ^2^ Dichotomous Thinking in Eating Scale; Cronbach alpha = 0.71; 2 items; Answer choices: 5-point agreement scale (i.e., strongly disagree, disagree, neither agree nor disagree, agree, strongly agree) [85]. ^3^ Food Neophobia Scale; Cronbach alpha = 0.83; 2 items; Answer choices: 5-point agreement scale (i.e., strongly disagree, disagree, neither agree nor disagree, agree, strongly agree) [86,87]. ^4^ Emotional Eating Scale; Cronbach alpha = 0.92; 5 items; Answer choices: 5-point descriptor scale (i.e., not at all, slightly, more or less, pretty well, completely) [71]. Stress-Induced Emotional Eating Subscale; Cronbach alpha = 0.88; 2 items. Depression-Induced Emotional Eating Subscale; Cronbach alpha = 0.89; 2 items. Relationship-Discord-Induced Emotional Eating Subscale; Cronbach alpha = n/a; 1 item. ^5^ Routine Restraint Scale; Cronbach alpha = 0.81; 3 items; Answer choices: 5-point descriptor scale (i.e., not at all, slightly, more or less, pretty well, completely) [71]. ^6^ Unhealthy Weight Control Methods Scale: Cronbach alpha = n/a; 9 items; Answer choices: yes/no. ^7^ Compensatory Restraint Scale; Cronbach alpha = 0.90; 3 items; Answer choices: 5-point descriptor scale (i.e., not at all, slightly, more or less, pretty well, completely) [71]. ^8^ Intuitive Eating Hunger & Satiety Scale; Cronbach alpha = 0.76; 3 items; Answer choices: 5-point agreement scale (i.e., strongly disagree, disagree, neither agree nor disagree, agree, strongly agree) [68]. ^9^ Mindful Eating Awareness Scale; Cronbach alpha = 0.75; 3 items; Answer choices: 5-point frequency scale (i.e., almost never, once in a while, sometimes, often, almost always) [69]. ^10^ Mindful Eating Attention Scale; Cronbach alpha = 0.69; 2 items; Answer choices: 5-point frequency scale (i.e., almost never, once in a while, sometimes, often, almost always) [69]. ^11^ 3-item scale; Cronbach alpha = n/a; 3 items; Days/week of walking, moderate activity, and vigorous activity, weighted by intensity levels of 1, 2, and 3, respectively, and summed to create scale score; scale scores range from 0 to 42; higher scale score indicates greater activity level.

**Table 3:** Binary Logistic Regression Analyses Examining Associations of Health Status and Weight-Related Behaviors with Body Dissatisfaction of Young Adult Women College Students (N = 210).

Characteristic	Body Satisfied # (*n* = 106) Mean ± SD	Body Dissatisfied (*n* = 104) Mean ± SD	SE †	Odds Ratio ‡ (95% Confidence Interval)	*p*-Value
Overall Health Status	3.60 ± 0.91	2.92 ± 1.00	0.158	0.48 (0.352–0.654)	<0.001
Physically Unhealthy Days	3.23 ± 4.36	5.43 ± 6.22	0.029	1.09 (1.025–1.149)	0.005
Mental Unhealthy Days	7.01 ± 7.36	10.83 ± 8.78	0.018	1.06 (1.024–1.099)	0.001
Body Mass Index	21.42 ± 2.39	24.87 ± 1.67	0.054	1.36 (1.220–1.507)	<0.001
Fruit/Vegetable Intake, servings/day	3.98 ± 2.07	3.81 ± 1.97	0.069	0.96 (0.838–1.097)	0.538
% Total Calories from Fat	33.23 ± 6.23	33.24 ± 5.24	0.000	1.00 (0.954–1.049)	0.995
Daily Sugar-Sweetened Beverage Intake, servings/day	0.53 ± 0.61	0.59 ± 0.64	0.222	1.16 (0.751–1.795)	0.502
Physical Activity Level	19.55 ± 12.90	17.62 ± 11.17	0.012	0.99 (0.965–1.009)	0.247
Sleep Duration, hours/night	6.81 ± 1.52	6.63 ± 1.73	0.087	0.93 (0.785–1.103)	0.408

# EDE-Q Body Shape Concerns question was used to classify those who were not at all or slightly dissatisfied as body satisfied and those who were moderately or a lot dissatisfied as body dissatisfied. † Standard Error. ‡ Binary logistic regression analyses examined separate associations of each characteristic with body dissatisfaction.

## Data Availability

No new data were created or analyzed in this study. Data sharing is not applicable to this article.
